# Supplementary material for: Analysis of common differential gene expression between rheumatoid arthritis and ulcerative colitis
Source: PLoS One. 2026 Jan 2;21(1):e0339397. doi: 10.1371/journal.pone.0339397 (PMC12758813; doi:10.1371/journal.pone.0339397)
Supplement: S1 Table — (DOCX) [file pone.0339397.s001.docx]

| Series ID | Platform ID | Tissue Source | Disese Samples | Control Samples | total |
| --- | --- | --- | --- | --- | --- |
| GSE77298 | GPL570 | synovial tissue | 16 | 7 | 23 |
| GSE12021 | GPL96 | synovial tissue | 12 | 9 | 21 |
| GSE55457 | GPL96 | synovial tissue | 13 | 10 | 23 |
| GSE89408 | GPL11154 | synovial tissue | 152 | 28 | 180 |
