# Supplementary material for: Analysis of common differential gene expression between rheumatoid arthritis and ulcerative colitis
Source: PLoS One. 2026 Jan 2;21(1):e0339397. doi: 10.1371/journal.pone.0339397 (PMC12758813; doi:10.1371/journal.pone.0339397)
Supplement: S2 Table — (DOCX) [file pone.0339397.s002.docx]

| Series ID | Platform ID | Tissue Source | Disese Samples | Control Samples | Total |
| --- | --- | --- | --- | --- | --- |
| GSE36807 | GPL570 | colon mucosal biopsy | 15 | 7 | 23 |
| GSE87473 | GPL13158 | colon mucosal biopsy | 106 | 21 | 127 |
| GSE92415 | GPL13158 | colon mucosal biopsy | 87 | 21 | 108 |
| GSE13367 | GPL570 | colon mucosal biopsy | 34 | 20 | 54 |
