# Supplementary figures and images for: Analysis of common differential gene expression between rheumatoid arthritis and ulcerative colitis
Source: PLoS One. 2026 Jan 2;21(1):e0339397. doi: 10.1371/journal.pone.0339397 (PMC12758813; doi:10.1371/journal.pone.0339397)

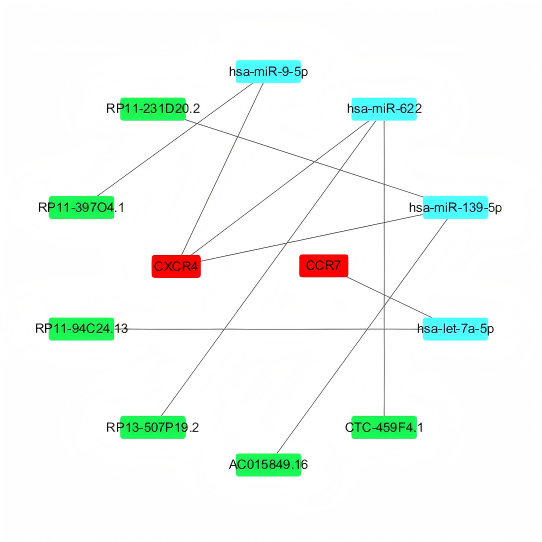

Supplement: S1 Fig — (TIF) [file pone.0339397.s017.tif]
