# Supplementary material for: Analysis of common differential gene expression between rheumatoid arthritis and ulcerative colitis
Source: PLoS One. 2026 Jan 2;21(1):e0339397. doi: 10.1371/journal.pone.0339397 (PMC12758813; doi:10.1371/journal.pone.0339397)
Supplement: Attachment — Submitted filename: PONE-D-24-53954.docx [file pone.0339397.s019.docx]

The study reports the Common Differential Gene Expression between Rheumatoid Arthritis (RA) and Ulcerative Colitis (UC) using datasets through bioinformatics analysis. However, there are key concerns which need to be addressed.

- The rationale for choosing these disease conditions RA and UC is not clear. Why did they did not choose other autoimmune conditions which are closely related to UC or RA.
- The reason for choosing these specific datasets is not clearly mentioned.
- Did they look at the other parameters like gender, age, treatment conditions and other existing co-morbidities which could influence the study outcomes.
- The results report the differentially expressed genes (DEG’s) such as CD19, CCR7 which are very common in other autoimmune conditions. The relevance of the DEG’s with other autoimmune disorders should be discussed in detail.
- The identified DEG’s should be validated experimentally to address the common molecular mechanisms.
- The quality of the figures should be improved, and the study can be strengthened by providing the detailed insights into relationship between these conditions.
