# Supplementary material for: Analysis of common differential gene expression between rheumatoid arthritis and ulcerative colitis
Source: PLoS One. 2026 Jan 2;21(1):e0339397. doi: 10.1371/journal.pone.0339397 (PMC12758813; doi:10.1371/journal.pone.0339397)
Supplement: Attachment — Submitted filename: Response to Reviewers.docx [file pone.0339397.s020.docx]

Dear Reviewers,

We wholeheartedly extend our gratitude to all of you for the precious feedback on our manuscript titled "Analysis of Common Differential Gene Expression between Rheumatoid Arthritis and Ulcerative Colitis". We are well aware of the immense effort and time you have devoted during the review process, and we hereby offer our sincere thanks. Your insightful remarks have not only enhanced our in-depth understanding of this research but also provided us with invaluable suggestions. We have carefully contemplated all the suggestions and will incorporate them one by one in the revised manuscript. Below are our point-by-point responses to each of your major comments.

Reviewer #1:

1. Dear Reviewer, I sincerely appreciate your invaluable review comments and your attention to enhancing the quality of this manuscript. Your observation regarding the "overstatement of therapeutic significance" is exceptionally insightful and of paramount importance. Indeed, it is scientifically rigorous to refer to the identified genes as "therapeutic targets" or "biomarkers" without functional validation, particularly for widely expressed genes like CD19. Following your suggestion, we have revised the relevant sections of the manuscript to present the potential research value of these genes in a more cautious manner.Terminology Revision: We have replaced the term "therapeutic targets" with the more prudent phrase "potential candidate genes for future research," emphasizing that these genes require further functional validation and clinical evidence before they can be confirmed as genuine therapeutic targets or biomarkers. Additionally, we have refined the language in certain paragraphs to prevent overinterpretation or exaggeration of the current study's conclusions.

2.Dear Reviewer,Thank you for reviewing my submission and providing your invaluable feedback. The issue you raised regarding the "lack of comparative analysis between rheumatoid arthritis and ulcerative colitis" is of critical importance. Although the primary objective of this study was to identify potential shared molecular mechanisms between RA and UC , as you correctly pointed out, the current research primarily focused on genes that were concurrently upregulated in both diseases, without systematically comparing their differential characteristics in terms of gene expression patterns, functional pathways, or pathological mechanisms. To better reflect the scientific value of this work, we have revised the research objectives to more precisely define the scope and focus. Specifically, the updated objectives now state:This study employs bioinformatics analysis with the objective of identifying commonly differentially expressed genes (DEGs) in ulcerative colitis (UC) and rheumatoid arthritis (RA), as well as exploring their underlying molecular mechanisms. By doing so, it aims to provide a theoretical basis for investigating the potential associations between these two diseases and developing novel therapeutic strategies.

3.Dear Reviewer,Thank you for reviewing my submission and providing your valuable feedback. In the section on "GENEMANIA online analysis," we listed ten hub genes and initially suggested that they might be involved in metabolic and thermogenic processes. However, later in the discussion section, we provided a more detailed description of these genes' functions, highlighting their primary associations with inflammatory responses and cell migration, rather than metabolic or thermogenic processes. We sincerely apologize for this inconsistency in our statements. To ensure the scientific rigor and logical coherence of the paper, we have revised the relevant sections. Specifically, we have re-examined and adjusted the descriptions in the "GENEMANIA online analysis" section to more accurately reflect the functional associations of these genes within the gene network. Furthermore, in the discussion section, we have further clarified the biological functions of these genes. For instance, we have removed the statement "involved in metabolic and thermogenic processes" and supplemented it with a more precise description: their central roles in various immune and inflammation-related biological processes, as well as their participation in immune cell activation, recruitment, and migration.

4.Dear Reviewer, I'm deeply grateful for your meticulous review of my submission and the precious feedback you've offered. We've conducted a systematic revision targeting language and structural issues: First off, we've gone through the manuscript sentence by sentence to rectify all spelling and grammatical errors. Next, we've disassembled and reconstructed lengthy and complex sentences, and employed logical connectors to enhance the coherence between paragraphs. Especially in the Methodology and Results sections, we've sharpened the content by trimming repetitive descriptions and consolidating similar information to boost its conciseness.

At the same time, we've re-examined and reorganized the scientific logical chain of the study. While keeping the core data intact, we've eliminated redundant expressions and supplemented key research details to enrich the completeness of the information.

In the Results section, we've annotated the full names for all abbreviations when they first appear in each paragraph. For the same abbreviations that show up later in the paragraph, we've uniformly used lowercase letters. Regarding unexplained abbreviations in the original manuscript, such as "Tph" and "WB," we've added their corresponding full phrases to ensure that readers can clearly comprehend the content.

5.Dear Reviewer,We have conducted a thorough inspection and optimization of the relevant figures and tables. We have adjusted their sizes and resolutions in accordance with the journal's requirements to ensure they meet the submission standards while maintaining both clarity and aesthetic appeal.

Reviewer #2: •

1.Dear Reviewer,Thank you for raising these significant questions. Regarding the selection of rheumatoid arthritis (RA) and ulcerative colitis (UC) as research subjects, we have made revisions in the manuscript based on the following scientific considerations:

(1) From an epidemiological perspective, both RA and UC have relatively high incidence rates globally, with a rising prevalence in recent years. Moreover, these two diseases exhibit a considerable overlap rate in clinical practice, particularly among UC patients, who have a significantly higher incidence of extraintestinal manifestations (such as arthritis) compared to patients with other intestinal diseases.

(2) In terms of pathological mechanisms, both RA and UC are chronic inflammatory diseases. RA primarily involves chronic inflammation of the joint synovium, while UC focuses on the inflammatory response in the intestinal mucosa. Despite their different lesion sites, both diseases are characterized by abnormal activation of the immune system and chronic inflammatory responses (e.g., Fcγ receptor signaling). Additionally, there is notable genetic overlap between RA and UC in the IL-23/Th17 pathway, providing an ideal comparative model for studying immune-related mechanisms.

(3) Research value and innovation: Selecting RA and UC as research subjects not only helps to uncover the common mechanisms underlying autoimmune diseases but also provides a theoretical basis for developing new therapeutic strategies. We have supplemented the research background section to more clearly explain the reasons for choosing RA and UC and emphasize their representativeness and scientific value in autoimmune disease research.

We highly value your revision suggestions and have made corresponding adjustments to the manuscript according to your feedback.

2. Dear Reviewer, The gene expression datasets we selected are all sourced from the public bioinformatics database—GEO (Gene Expression Omnibus). GEO is a high-throughput gene expression data repository maintained by the National Center for Biotechnology Information (NCBI) in the United States. It encompasses a wide range of research fields and features high-quality data with a broad clinical application background. The datasets we chose include samples from patients with rheumatoid arthritis (RA) and ulcerative colitis (UC), as well as healthy control groups, with each group comprising more than 20 samples. This sample size ensures the statistical significance of our experimental results and helps avoid phenotypic confounding due to insufficient sample sizes. Additionally, we prioritized datasets that align with our specific research objectives to ensure data comparability and the reliability of our analyses.

3.Dear Reviewer, Thank you for your questions. We hereby provide the following responses to the issues you raised: We explicitly acknowledge that parameters such as age, gender, treatment conditions, and comorbidities may potentially confound gene expression profiles and serve as important background variables in certain analyses. However, due to limitations in the data collection process, we were unable to obtain detailed age group and gender information for all samples. Given that our primary research objective is to minimize batch effects across datasets through rigorous normalization procedures, identify differentially expressed genes associated with rheumatoid arthritis and ulcerative colitis, and explore the roles of these genes in disease progression, we decided to include the aforementioned datasets in our study and performed standardization processes during subsequent data analysis. We believe that this approach can, to a certain extent, balance the influence of confounding factors while focusing on the core objectives of our research. This aspect has been clarified in the "Limitations" section of our article.

4.Dear Reviewer,Thank you for this valuable suggestion. Delving into the performance of these differentially expressed genes (DEGs) in other autoimmune diseases will indeed facilitate a better understanding of the potential significance of our findings. In response to your advice, we have added supplementary discussions in the relevant section. We believe that these additional analyses have substantially enhanced the depth and breadth of our study, enabling readers to gain a more comprehensive understanding of the disease-specific and shared characteristics of these DEGs.

5.Dear Reviewer,We sincerely appreciate your valuable feedback. We have come to deeply recognize the pivotal role that experimental validation plays in research. In this study, we conducted preliminary validation using an additional dataset. Given the large number of genes initially screened, validating each one individually would lack focus and efficiency. Therefore, we plan to further screen for 1 - 3 ideal genes through the construction of animal models in the later stage for subsequent validation. However, constructing and validating animal models takes time. Thus, we intend to report the above experimental content and validation results in a forthcoming paper.

The current research findings serve as the preliminary results for subsequent experiments. Nevertheless, it is undeniable that they lack the support of replicable experimental results. Hence, we have added a detailed explanation of this limitation and our future prospects in the "Limitations" section of the manuscript.Once again, we thank you for your precious and insightful suggestions, which have been of immense help in improving our research and enhancing its scientific quality.

6. Dear Reviewer,Regarding the issue of the images, we have utilized the official PLOS ONE processing platform PACE (https://pacev2.apexcovantage.com/ Home Page - Apex.PACE ) to adjust the dimensions and resolution of the charts and graphs. This ensures that they meet the submission requirements while maintaining both clarity and aesthetic appeal.As for the detailed explanation you requested concerning the relationship between rheumatoid arthritis (RA) and ulcerative colitis (UC), we have made supplementary enhancements in the revised manuscript, primarily based on the following considerations: We explored the connections between the extra-articular manifestations of RA and UC. In terms of immune pathways, both diseases are characterized by abnormal activation of the immune system and chronic inflammatory responses. Additionally, there is a significant genetic overlap in the IL-23/Th17 pathway between the two. Moreover, they share common genetic and molecular mechanisms. We delved into the genetic overlaps between RA and UC, such as the differential expression of genes like CCR7, CD19, CXCL13, CXCR4, and SELL, and their roles in the diseases.However, it is important to note that current research still requires stronger experimental validation to confirm the practical clinical value of the identified genes and pathways.

We again thank the reviewer for their valuable comments and look forward to your further feedback on our revised manuscript.

Best regards,

Wei-rong Cui
