# Supplementary material for: Analysis of common differential gene expression between rheumatoid arthritis and ulcerative colitis
Source: PLoS One. 2026 Jan 2;21(1):e0339397. doi: 10.1371/journal.pone.0339397 (PMC12758813; doi:10.1371/journal.pone.0339397)
Supplement: Attachment — Submitted filename: Response to Reviewers .docx [file pone.0339397.s021.docx]

Dear Reviewers,

We sincerely appreciate the valuable comments and suggestions regarding our manuscript entitled "Differential Gene Expression Analysis of Common Signatures Between Rheumatoid Arthritis and Ulcerative Colitis." We are truly grateful for the considerable time and effort you have dedicated to the review process. Your insightful critiques have not only deepened our understanding of this study but have also provided us with invaluable recommendations. We have carefully considered each comment and will address them point by point in the revised version. Below are our detailed responses to your primary concerns.

1. We sincerely thank the reviewer for this valuable comment. The reviewer rightly pointed out that integrating datasets from different platforms in validation studies introduces potential batch effects as a critical issue, which may introduce technical biases and lead to false positive findings. We fully agree with this perspective and have prioritized this consideration in our analytical process. Prior to the integrative analysis of validation datasets from different platforms, we specifically applied the ComBat algorithm from the R package "sva" to correct for batch effects in the gene expression matrix. This method is widely recognized as a gold standard for addressing batch effects in genomic data, effectively identifying and removing non-biological variations introduced by technical differences across experimental platforms. Following batch effect correction, we performed differential expression analysis using the R package "limma". Limma accounts for various sources of variation within linear models, thereby yielding more accurate statistical inferences. For the validation phase, our approach primarily involved conducting differential expression analysis within each independent validation dataset to assess whether our core findings could be consistently replicated across different platforms and cohorts. This cross-platform reproducibility substantially reduces the likelihood of false positives and strongly supports the robustness of our conclusions.
2. We sincerely thank you for raising this important and insightful point. You correctly noted that the GSE92415 dataset, derived from a golimumab clinical trial, includes paired samples collected both before and after treatment, and that the pharmacological intervention could significantly alter gene expression profiles. We fully agree that carefully accounting for treatment status is crucial in the analytical design.Upon careful re-examination, we confirm that our initial analysis did not adequately distinguish between pre- and post-treatment samples within the GSE92415 dataset, instead analyzing all samples collectively, which may have introduced confounding bias due to drug effects. We have promptly reanalyzed the data, utilizing only the pre-treatment (baseline) samples for all relevant analyses.We are pleased to report that the final gene list obtained from this reanalysis remains consistent with our original findings derived from the full dataset. This outcome not only indicates the relative stability of the gene signatures we initially identified but also validates the effectiveness of the corrective measures taken during the reanalysis, thereby enhancing the reliability and accuracy of our results.Once again, we deeply appreciate your attentive review and guidance. Your expert comments have been instrumental in further refining our study and improving the overall quality of our work.
3. We sincerely thank you for reviewing our manuscript and providing this valuable feedback. You rightly pointed out the lack of clarity in the description of our data analysis workflow and the confusion regarding the methods used—a critical issue with which we fully concur. We acknowledge that this was a significant oversight in our manuscript, which compromised the clarity and reproducibility of our methods, and we sincerely apologize for this error.Upon carefully re-examining our analysis code and records, we confirm that the limma method was in fact used for differential gene expression analysis of the microarray data in this study. The limma package is suitable for analyzing gene expression data generated by either microarray or RNA-seq technologies. The mention of "DESeq2" in the Methods section was an unfortunate typographical error; while DESeq2 is typically applied to RNA-seq data, it was not used in our study. We take full responsibility for this confusion between the two methodological names.We have now completely removed all incorrect references to "DESeq2" and have thoroughly revised the Methods section to accurately and clearly describe the analytical workflow.
4. We sincerely thank you for this important and insightful comment. You have rightly pointed out the lack of clarity in our methodological description, particularly regarding the need for tailored analytical workflows for different data types. We fully agree that precise description of analytical methods is crucial for research reproducibility.Upon careful re-examination, we confirm that the limma package was consistently used for all differential expression analyses during the validation phase. The limma package is well-suited for analyzing gene expression data generated by either microarray or RNA-seq technologies, which ensured methodological consistency throughout our study.
5. We sincerely thank you for this important and expert comment. You correctly highlighted the risk that batch effect correction may potentially remove genuine biological signals, as well as the confounding influence of cellular composition on result interpretation. We fully agree that these methodological considerations are crucial for ensuring the reliability of our findings.Following your suggestion, we performed comprehensive PCA visualization analysis. We have now included PCA plots grouped by "Case/Control" status alongside those grouped by "Experimental Batch". These results clearly demonstrate that ComBat correction effectively removed technical variations across batches while successfully preserving and even highlighting the biological differences between the case group (triangles) and the control group (circles).Furthermore, we employed the CIBERSORT algorithm with its LM22 signature matrix to accurately estimate the proportions of 22 immune cell subtypes within each sample.
6. Thank you for pointing out this key terminology error. You are absolutely correct. In our study, we employed the Maximal Clique Centrality (MCC) method from the CytoHubba plugin to identify hub nodes within the network. We sincerely apologize for the typographical error in our original description, where it was mistakenly referred to as "modularity class," which is indeed inaccurate. This error has been comprehensively corrected throughout the revised manuscript.
7. Thank you for your attention to our analytical methodology. We selected a correlation threshold of 0.4 for network construction, which represents a widely accepted and moderately stringent cutoff in the field. This choice was based on the need to balance network scale with connection specificity: an excessively low threshold (e.g., 0.2) would introduce numerous weak or false-positive connections, resulting in an overly dense network, while an overly high threshold (e.g., 0.6) could exclude biologically meaningful signals, leading to an excessively sparse network. The threshold of 0.4 was chosen to retain meaningful biological associations while minimizing biases associated with overly lenient or stringent cutoffs. This threshold is further supported by its application in several published studies, such as:(Zhang R, Lan X, Zhu W, Wang L, Liu P, Li P. Regulation of autophagy by the PI3K-AKT pathway in Astragalus membranaceus–Cornus officinalis to ameliorate diabetic nephropathy. Front Pharmacol. 2025;16:1505637. Liu H, Wu M, Qi G, Ma F, Cao Y. Identification and Validation of Potential Diagnostic Biomarkers for Pulmonary Arterial Hypertension Based on Gene Expression Profiling. Pulm Circ. 2025;15(4):e70207.) To evaluate the robustness of the identified hub genes, we systematically varied the correlation threshold from 0.3 to 0.5 and re-identified hub genes under each condition. The results demonstrated that the top-ranked core hub genes remained largely stable across this range of thresholds. This confirms that these core hubs are not artifacts of a specific threshold value but represent intrinsic key nodes within the network structure.

Understanding the subcellular localization of hub genes is essential for interpreting their biological functions. As suggested, we performed a detailed compartment-based analysis using the Human Protein Atlas (HPA) database, which yielded important insights: the five hub genes exhibit a clear localization dichotomy, with 80% (SELL, CCR7, CD19, and CXCR4) being membrane-localized proteins primarily positioned on the plasma membrane, while the remaining 20% (CXCL13) is a secreted protein localized to the extracellular space. Based on these localization patterns, we propose a plausible functional divergence: the membrane proteins are likely involved in immune recognition and recruitment through direct cell-surface interactions, whereas the secreted protein CXCL13 may modulate the microenvironment via paracrine signaling. This spatial perspective provides a compelling explanation for their distinct immune-correlation patterns.

Tissue heterogeneity and the confounding effects of immune cell infiltration are critical issues that must be seriously considered. To directly quantify and address this problem, we systematically performed immune infiltration association analysis on the identified hub genes using the CIBERSORT algorithm. This analysis provided key evidence to distinguish whether changes in gene expression originate from differences in immune cell composition or from pathological programs of tissue-resident cells.

Our analysis yielded clear and discriminative conclusions: In UC, we observed significant heterogeneity in immune infiltration. Through correlation analysis, we successfully categorized the hub genes into two groups: one group (e.g., CD19, CXCL13) showed strong correlations with the abundance of adaptive immune cells (such as B cells and follicular helper T cells), indicating that their hub status is partially driven by immune cell infiltration; whereas the other group (e.g., CXCR4) remained relatively independent of immune cell variations, suggesting that it is more likely to reflect intrinsic pathological processes of intestinal resident cells. In RA, we discovered a distinctly different pattern. Immune infiltration in synovial tissue showed high consistency across samples, and more importantly, none of the hub genes (SELL, CCR7, CD19, CXCL13, CXCR4) showed significant correlations with the proportions of any immune cell type. This key evidence strongly indicates that in RA, the expression changes of our identified hub genes are not confounded by variations in immune cell composition among samples, but rather represent core pathogenic molecular programs that are stably activated within resident synovial cells during disease progression.We have systematically integrated these key analyses and conclusions into the manuscript and included the immune cell correlation results in the supplementary files. We once again thank you for your insightful comments, which have helped us better highlight the novelty and rigor of our study. We hope the current revisions meet your requirements.

8. We sincerely thank you for these rigorous and insightful comments. Your request for detailed statistical reporting and robustness validation is crucial for ensuring the reliability of our findings. In accordance with your suggestions, we have conducted comprehensive supplementary analyses, as detailed below: We have calculated and plotted ROC curves for each validated gene (Figure 10), and reported the log2FC (with 95% CI) and Benjamini-Hochberg (BH) adjusted p-values. These results have been compiled in a supplementary table included in the supplementary files. Furthermore, we rigorously performed a "leave-one-dataset-out" study-level validation. The results demonstrate that our identified hub genes remained statistically significant across all validation rounds, with stable AUC values, confirming the exceptional robustness of our findings.

9.Sincerely thank you for this rigorous and highly constructive feedback. You correctly pointed out that, in the absence of cross-cohort co-expression consistency validation, ceRNA analysis based on bioinformatic predictions is more appropriately framed as hypothesis-generating rather than as definitive evidence. We fully agree with this perspective. To ensure the robustness of our paper’s conclusions and adhere to the highest standards of scientific rigor, we have moved the ceRNA network figure from the main text to the supplementary materials. In both the Results and Methods sections of the manuscript, we have revised all relevant descriptions to clearly emphasize the exploratory and hypothesis-generating nature of this analysis, and have removed any language that could be interpreted as implying causal relationships or overstating the findings.

10.We sincerely thank you for this important comment. We fully agree that directly inferring "potential therapeutic targets" based on cross-sectional gene expression data and network centrality analysis constitutes an overinterpretation of the clinical implications and incorrectly implies causality. We have thoroughly revised the Discussion section to address this issue.

11. We thank the reviewer for this suggestion, which helps enhance the reproducibility of our work. In accordance with this request, we have included all requested materials in the supplementary files.

We again thank the reviewer for their valuable comments and look forward to your further feedback on our revised manuscript.

Best regards,

Wei-rong Cui
